# Reproductive decision-making in the context of hereditary cancer: the effects of an online decision aid on informed decision-making

**DOI:** 10.1007/s12687-020-00484-2

**Published:** 2020-09-02

**Authors:** Kelly Reumkens, Marly H. E. Tummers, Yil Severijns, Joyce J. G. Gietel-Habets, Sander M. J. van Kuijk, Cora M. Aalfs, Christi J. van Asperen, Margreet G. E. M. Ausems, Margriet Collée, Charlotte J. Dommering, Marleen Kets, Lizet E. van der Kolk, Jan C. Oosterwijk, Vivianne C. G. Tjan-Heijnen, Trudy van der Weijden, Christine E. M. de Die-Smulders, Liesbeth A. D. M. van Osch

**Affiliations:** 1 Department of Clinical Genetics, Maastricht University Medical Centre+, Maastricht, the Netherlands; 2 GROW School for Oncology and Developmental Biology, Maastricht University Medical Centre+, Maastricht, the Netherlands; 3 Department of Health Promotion, School CAPHRI, Faculty of Health, Medicine and Life Sciences, Maastricht University, Postbox 616, 6200 MD Maastricht, the Netherlands; 4 Department of Clinical Epidemiology and Medical Technology Assessment, Maastricht University Medical Centre+, Maastricht, the Netherlands; 5 Department of Clinical Genetics, Amsterdam UMC, Academic Medical Centre, Amsterdam, the Netherlands; 6 Department of Clinical Genetics, Leiden University Medical Center, Leiden, the Netherlands; 7 Department of Genetics, Division of Biomedical Genetics, University Medical Center Utrecht, Utrecht, the Netherlands; 8 Department of Clinical Genetics, Erasmus University Medical Center, Rotterdam, the Netherlands; 9 Department of Clinical Genetics, Amsterdam UMC, Vrije Universiteit Amsterdam, Amsterdam, the Netherlands; 10 Department of Human Genetics, Radboud University Medical Center Nijmegen, Nijmegen, the Netherlands; 11 Family Cancer Clinic, Netherlands Cancer Institute, Amsterdam, the Netherlands; 12 Department of Genetics, Groningen University Medical Center, University of Groningen, Groningen, the Netherlands; 13 Department of Medical Oncology, Maastricht University Medical Centre+, Maastricht, the Netherlands; 14 Department of Family Medicine, School CAPHRI, Faculty of Health, Medicine and Life Sciences, Maastricht University, Maastricht, the Netherlands

**Keywords:** Decision aid, Hereditary cancer, Patient participation, Preimplantation genetic testing, Prenatal diagnosis

## Abstract

Individuals having a genetic predisposition to cancer and their partners face challenging decisions regarding their wish to have children. This study aimed to determine the effects of an online decision aid to support couples in making an informed decision regarding their reproductive options. A nationwide pretest-posttest study was conducted in the Netherlands among 131 participants between November 2016 and May 2018. Couples were eligible for participation if one partner had a pathogenic variant predisposing for an autosomal dominant hereditary cancer syndrome. Participants completed a questionnaire before use (T0), and at 3 months (T3) after use of the decision aid to assess the primary outcome measure informed decision-making, and the secondary outcome measures decisional conflict, knowledge, realistic expectations, level of deliberation, and decision self-efficacy. T0–T3 comparisons show an overall positive effect for all outcome measures (all *p*s < 0.05; knowledge (ES = − 1.05), decisional conflict (ES = 0.99), participants’ decision self-efficacy (ES = −0.55), level of deliberation (ES = − 0.50), and realistic expectations (ES = − 0.44). Informed decision-making increased over time and 58.0% of the participants made an informed reproductive decision at T3. The online decision aid seems to be an appropriate tool to complement standard reproductive counseling to support our target group in making an informed reproductive decision. Use of the decision aid may lessen the negative psychological impact of decision-making on couples’ daily life and wellbeing.

## Background

Decisional support strategies (e.g., use of decision aids) are designed to help patients in making specific and deliberative choices regarding their health(care) and can be effective in promoting informed and shared decision-making (ISDM) (O’Connor and Jacobsen 2003; Juraskova et al. 2014; Stacey et al. 2017). Patient decision aids are particularly useful to support patients in decision-making regarding choices that are preference-sensitive and value-laden. These characteristics are exemplary for reproductive decision-making among persons with an autosomal dominant predisposition for hereditary cancer and their partners, as they are faced with a 50% risk of transmitting the pathogenic variant in one of the cancer genes to their offspring.

Three reproductive options are available for couples who strive for a child that is genetically related to both partners: (1) natural conception without genetic testing, implying acceptance of the risk of passing on the pathogenic variant to offspring; (2) prenatal diagnosis (PND), with the possibility to terminate the pregnancy if the fetus is affected by the pathogenic variant; and (3) preimplantation genetic testing (PGT), offering couples the option to obtain embryos by in vitro fertilization (IVF) and test them for the familial pathogenic variant. Only embryos without the pathogenic variant are transferred into the uterus. PGT is a physically intensive and relatively lengthy trajectory with a chance of an ongoing pregnancy of approximately 25% per treatment (De Rycke et al. 2015). However, PGT circumvents the emotional and physical burden of a pregnancy termination after prenatal testing (de Die-Smulders et al. 2013). PGT and PND are common for hereditary cancer; PGT for hereditary cancer predispositions is possible in the Netherlands since 2008. Since this legalization in the Netherlands, PGT for hereditary breast and ovarian cancer evolved into one of the most often applied PGT indications. PND is also possible for some types of hereditary cancer but is used far less than PGT. During counseling, most couples of reproductive age receive information on the PGT and PND procedures (PGD Nederland 2018).

Previous studies among couples who are aware that one of both partners has a genetic predisposition to cancer and who have a high genetic risk of transmitting this predisposition to their offspring, indicated that couples experience difficulties with reproductive decision-making (Derks-Smeets et al. 2014; Gietel-Habets et al. 2018; Dekeuwer and Bateman 2013; Ormondroyd et al. 2012; Dommering et al. 2010; Donnelly et al. 2013; Van Asperen et al. 2002). Couples have to cope with the increased risk of developing cancer of one of the partners and additionally are worried about the risk of passing on the predisposition to cancer to their offspring (Smith et al. 2004). Different physical, psychological, social, moral/ethical, and practical motives and considerations are taken into account when deciding on a reproductive option, which makes this a challenging process. The decision can have a major emotional impact as couples may experience feelings of guilt or doubt up to years after making their decision (Derks-Smeets et al. 2014). Incorporating an online decision aid in the reproductive decision-making process may optimize the quality of the reproductive decision itself, as well as improve the overall reproductive decision-making process (Derks-Smeets et al. 2014; Quinn et al. 2010).

The present study is part of a larger project on the development and implementation of an online decision aid with the aim of supporting couples who are aware that one of them has a genetic predisposition to cancer, to decide on the personally most suitable reproductive option (Reumkens et al. 2018; Reumkens et al. 2019a). In a previous study, short-term effects of the online decision aid in supporting informed reproductive decision-making among our target group were investigated (Reumkens et al. 2019b). In this study, we report on the effects on informed decision-making by both partners.

## Methods

A nationwide pretest-posttest study was conducted in the Netherlands between November 2016 and May 2018 among persons who have a genetic predisposition to cancer and their partners with a wish to have children.

### Participants and procedures

Eligible couples were recruited during or after oncogenetic consultation in one of the Clinical Genetics Departments in the Netherlands (i.e., all nine departments participated in the study). Healthcare providers involved in the oncogenetic counseling (i.e., clinical geneticists, genetic counselors, and social workers) recruited couples. Couples were eligible for participation if one partner had a pathogenic variant predisposing for an autosomal dominant hereditary cancer syndrome for which the reproductive options (PND/PGT) are available in the Netherlands. Furthermore, couples had to have the intention to try to conceive within 5 years, had to be 18 years or older, had not yet made a decision regarding their preferred reproductive option, and needed to have sufficient knowledge of the Dutch language.

Couples were provided with an information brochure by their healthcare provider, including a link to an online registration page, with an explanation of the study procedure. Although participation of both partners was encouraged, participation of one partner (regardless of being carrier) was allowed. After registration, both partners received an informed consent form by e-mail. After providing online informed consent, both partners were individually directed to an online baseline questionnaire (T0). They received a personal login code to access the decision aid after completing the baseline questionnaire. Questionnaires were completed separately by both partners but it was allowed to use the decision aid together. Immediately after use (T1), 2 weeks (T2), and 3 months after use of the decision aid (T3), participants were again directed to an online questionnaire. In this paper we will compare outcome measures at T0 and T3, comparisons of T0–T1 and T0–T2 are described elsewhere (Reumkens et al. 2019b). Findings regarding the outcome informed decision-making could not be compared to T1 and T2 since not all concepts of informed decision-making (knowledge, deliberation, and value consistency) were assessed at T1 and T2. A reminder was sent to participants who did not complete T3 within 3 weeks after invitation. After completion of the study, participants received an incentive of 15 euros in vouchers.

### Content of the decision aid

For an explanation of the developmental process and the content of the online decision aid, see Reumkens et al. 2018 (Reumkens et al. 2019a). In short, the decision aid contained:

1. Information about the risk of transmitting the pathogenic variant to their offspring and couples’ reproductive options to have genetically related children.
2. Treatment burden of reproductive options and the chances of different outcomes (e.g., risk of miscarriage after PND) presented in different formats using text and videos (e.g., verbal, and population diagrams) (Reumkens et al. 2018; Trevena et al. 2013).
3. An option grid of important features of each option.
4. Value clarification exercises (VCE) (Fagerlin et al. 2013). The VCE includes 18 statements representing values and motives considered important for reproductive decision-making (Derks-Smeets et al. 2014).
5. The possibility to generate a combined overview of both partners’ responses on the VCE.
6. A question prompt sheet, providing examples of questions and requests for additional information and space for own questions.
7. Information regarding the scientific resources used to underpin the content of the DA, information on the development team, funding, and contact information.

### Questionnaires

Baseline characteristics assessed at T0 were gender, age, educational level, carrier status, disease type, history of cancer in the at risk person, reproductive history, number of children, and future planning to have children. Couples were furthermore asked if they already had had a consultation with a healthcare provider in which the reproductive options were discussed.

The primary outcome measure was informed decision-making, and secondary outcome measures were decisional conflict, knowledge of the reproductive options, realistic expectations, level of deliberation, and decision self-efficacy. An overview of the content of the questionnaires is provided in Table 1.

**Table 1:** Questionnaires

	Questionnaire	Number of items	Answering scale	Scoring	Cronbach’s *α*
Decisional conflict	Decisional Conflict Scale (O’Connor 1995).	16	0 (strongly agree)–4 (strongly disagree)	0–100	0.85
Decisional conflict excl. the effective decision subscale*	Decisional Conflict Scale (O’Connor 1995).	12	0 (strongly agree)–4 (strongly disagree)	0–100	0.91
Knowledge of the three reproductive options	Gietel-Habets et al. 2017 (Gietel-Habets et al. 2017).	15	1 = correct, 2 = incorrect, 3 = not sure (recoded into 0 = incorrect, 1 = correct)	0–15	-
Realistic expectations	Realistic Expectations Scale (O’Connor 2002).	3	Variable (recoded into 0 = incorrect, 1 = correct)	0–3	-
Level of deliberation	Deliberation Scale (Van den berg et al. 2006).	6	1 (totally disagree)–5 (totally agree)	6–30	0.91
Decision self-efficacy	Decision Self-Efficacy Scale (Bunn and O’Connor 1996).	11	0 (not at all confident)–4 (very confident)	0–100	0.90
Attitude regarding the reproductive options	Attitude Scale (Marteau et al. 2001).	4 per reproductive option (total 12)	1 (e.g., beneficial and pleasant)–7 (e.g., harmful and unpleasant)	4–28	-

*As the items in the effective decision subscale from the Decisional Conflict Scale could not be completed by couples who did not have a preferred reproductive option in mind, a combined score was also calculated for the 4 other subscales. These 12 items were summed, divided by 12, and multiplied by 25. Total scores ranged from 0 (no decisional conflict) to 100 (extremely high decisional conflict).

Informed decision-making was measured at T0 and T3 by analyzing the outcomes knowledge, deliberation, and attitude towards the reproductive options (Van den Berg et al. 2006; Marteau et al. 2001). A reproductive decision was considered to be informed if a participant had a sufficient knowledge level, a high level of deliberation, and if the reproductive option was value-consistent (Van den Berg et al. 2006). To measure informed decision-making, knowledge and deliberation were recoded into dichotomous variables. Based on Abbott’s formula, knowledge levels of ≥ 12 (total 15) were considered to be sufficient (Frary 1988). Furthermore, based on median scores, deliberation scores of ≥ 19 (total 30) were considered to be high levels of deliberation. Participants’ (preferred) reproductive option was classified as value-consistent if participants had a positive attitude (score ≥ 15: total 28) towards this option. It should be noted that behavioral implementation or adherence to the reproductive decision (i.e., uptake of the test (PND) or treatment (PGT)) is unlikely to have occurred for all couples during the study period. Therefore, it is only possible to measure informed choice as defined by Marteau et al. 2001 (i.e., an informed choice is one that is based on relevant knowledge, consistent with the decision maker’s values and behaviorally implemented) for couples who reported at T3 that they try to conceive or started the PGT trajectory (Marteau et al. 2001).

### Statistical analyses

Data from the baseline characteristics were analyzed by means of descriptive statistics and quantified as mean and standard deviation or absolute number and percentage. To decide what statistical model could best be used to analyze data that are clustered both within participants (i.e., before–after) and within couples, we compared two models: one linear mixed-effects model in which clustering within participants over time and within couples was corrected for, and one model without correction for clustering within couples. Both models yielded similar results. A likelihood-ratio test showed that correction for the clustering of observations within couples did not lead to a better model fit (likelihood ratio = 0.00, *p* = 1.000). Since it was sufficient to take the within-participant clustering into account, paired sample *t* tests were conducted to compute differences between the first and subsequent measurement for the continuous variables. To assess the effects of the decision aid for several subgroups, we first split our sample into two groups (e.g., males and females) before conducting a paired sample *t* test for each group. An exact McNemar’s test was performed to assess the effects of the decision aid on informed decision-making. Cohen’s *d* was used to report effect sizes; an effect size quantifies the strength of an effect between group means. Analyses were performed using IBM SPSS version 23 and R version 3.3.3. *p* values less than 0.05 were considered significant.

## Results

### Baseline characteristics

T0 was completed by 131 participants and 93 participants completed T3 (29.0% dropout). At T0, 122 participants were part of a couple; nine participants took part without their partner. Over half of the participants were female (55.0%). Males were slightly older compared with females (32.3 vs. 29.6 years). More than half of the participants had a high education level (57.3%). The most frequently reported hereditary cancer syndrome was hereditary breast and ovarian cancer (HBOC) (84.7%). The majority (73.3%) indicated that they wanted to have children within 2 years and 16.8% already had offspring. The majority of the participants (77%) already had a consultation in which the reproductive options were discussed. An overview of participants’ baseline characteristics is provided in Table 2.

**Table 2:** Participants’ characteristics at baseline

	Sample (%) (*n* = 131)
Gender	
Male	59 (45.0)
Female	72 (55.0)
Age (years)	
Male	32.3 (SD = 3.7)
Female	29.6 (SD = 3.3)
Education^1^	
Low/middle	56 (42.7)
High	75 (57.3)
Carrier status	
Male carrier	40 (30.5)
Female carrier	91 (69.5)
Hereditary cancer types	
Hereditary breast and ovarian cancer	112 (85.5)
Hereditary nonpolyposis colorectal cancer/lynch syndrome	10 (7.6)
Familial adenomatous polyposis	2 (1.5)
Familial atypical multiple mole/melanoma syndrome	1 (0.8)
Li-Fraumeni syndrome	2 (1.5)
Hereditary diffuse gastric cancer	2 (1.5)
Hereditary leiomyomatosis and renal cell carcinoma	2 (1.5)
Have (had) cancer	
Yes	18 (13.7)
No	113 (86.3)
Received counseling	
Yes	101 (77.1)
No	30 (22.9)
Reproductive history	
Children	
Yes	22 (16.8)
No	109 (83.2)
Planning to have children	
Trying to conceive	13 (9.9)
Plan to have children within two years	83 (63.4)
Plan to have children within five years	29 (22.1)
Maybe, not sure yet	4 (3.1)
Otherwise	2 (1.5)
Preferred reproductive option	
Natural conception without genetic testing	31
PND	2
PGT	82
I don’t know	15
Missing	1

^1^In 15 couples, both partners were low educated

### Effects of the decision aid

#### Primary outcome measure: informed decision-making

**Fig. 1:**
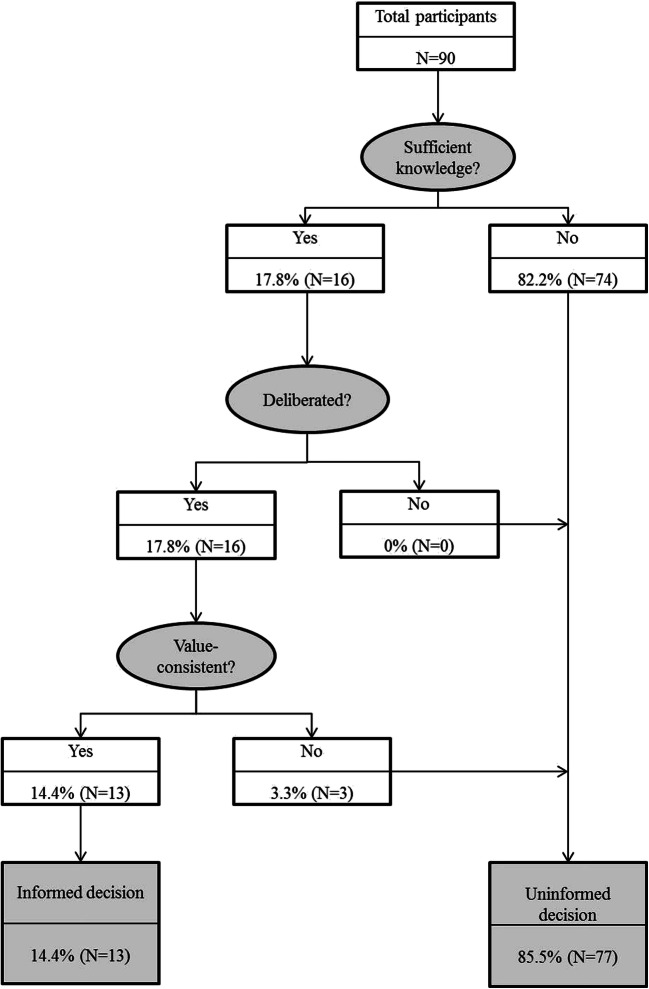
Informed decision-making T0

For 14.4% of the participants, the reproductive decision (i.e., the preferred reproductive option, e.g., PGT) at baseline met the conditions for informed decision-making (Fig. 1). Only 17.8% of the participants made a knowledge-based decision, 17.8% made a decision that was knowledge-based *and* deliberated, and 14.4% made a decision that was knowledge-based *and* deliberated *and* value-consistent, thereby meeting all three prerequisites for informed decision-making at baseline. As the items of the Deliberation Scale, essential to measure informed decision-making, were not fully completed by all couples, we were able to include a total of 90 participants at baseline and 69 participants at T3.

**Fig. 2:**
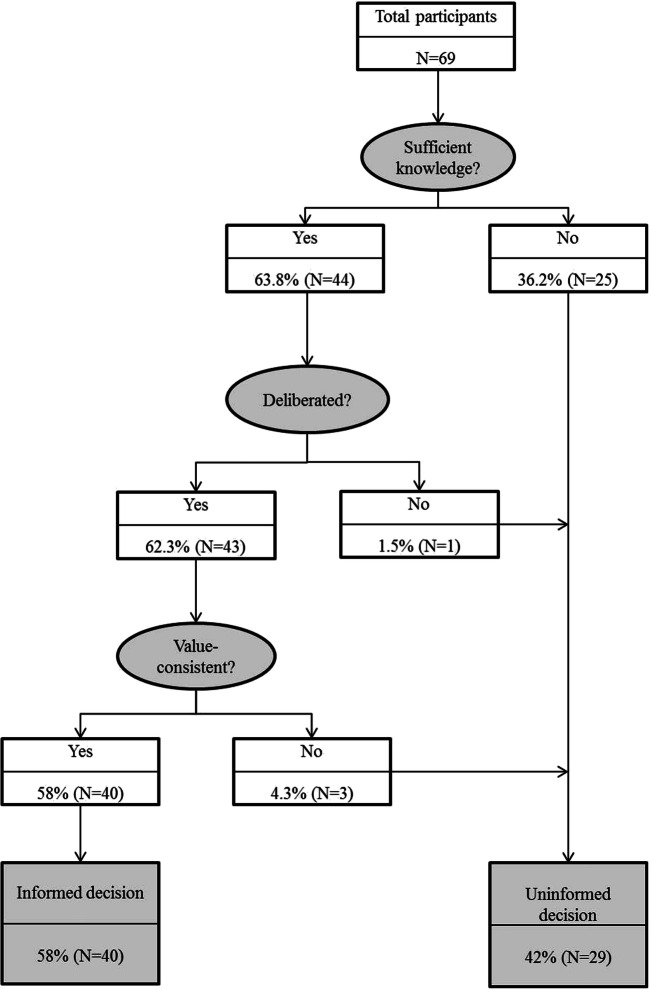
Informed decision-making T3

Informed decision-making significantly increased over time (*p* < 0.001). At T3, 63.8% of the participants made a knowledge-based decision, 62.3% made a decision that was knowledge-based *and* deliberated, and 58.0% made a decision that was knowledge-based *and* deliberated *and* value-consistent, and therefore meeting all three prerequisites for informed decision-making (Fig. 2).

Informed decision-making regarding the reproductive options was furthermore assessed at the couple level (i.e., both partners of a couple made an informed reproductive decision) and significantly increased over time (*p* < 0.001). A total of 76 participants participated as a couple at T0 (38 couples). In 26 of these 38 couples (68.4%), both partners had the same preferred reproductive option. Only two out of these 26 couples met the conditions for informed decision-making for both partners at baseline (5.3% of total; 7.7% of couples agreeing on the same reproductive option). A total of 58 participants participated as a couple at T3 (29 couples). In 25 of 29 couples (86.2%), both partners preferred the same reproductive option. Ten out of these 25 couples met the conditions for informed decision-making for both partners (34.5% of total; 40.0% of couples agreeing on the same reproductive option). Nineteen out of these 25 couples (76.0%) behaviorally implemented their reproductive decision at T3 (i.e., trying to conceive or started the PGT trajectory). Seven of these 19 couples met the conditions for informed decision-making for both partners at T3 (24.1% of total couples; 36.8% of couples who implemented their decision (e.g., starting with PGT trajectory).

#### Secondary outcome measures

The decision aid showed significant effects for all secondary outcome measures. The largest effect size was found for knowledge (ES = − 1.05), followed by decisional conflict (ES = 0.99), participants’ decision self-efficacy (ES = − 0.55), level of deliberation (ES = − 0.50), and realistic expectations (ES = − 0.44) (Table 3).

**Table 3:** Effects of decision aid on outcome measures

	M (SD) T0	M (SD) T3	*T*	*p*
Decisional conflict				
Total score (0–100)*	28.16 (10.26)	14.16 (11.10)	6.83	< 0.001
Total score (excl. effective decision; 0–100)	36.97 (18.72)	29.21 (26.19)	2.21	0.029
Uncertainty subscale	49.68 (26.57)	28.46 (23.07)	8.06	< 0.001
Informed subscale	34.42 (19.98)	16.02 (15.40)	7.50	< 0.001
Values clarity subscale	37.34 (23.40)	16.99 (15.02)	7.66	< 0.001
Support subscale	33.66 (17.49)	18.51 (16.37)	7.16	< 0.001
Effective decision subscale*	23.70 (17.49)	11.20 (12.56)	4.19	< 0.001
Knowledge				
Total score (0–15)	9.45 (2.80)	12.33 (1.78)	− 9.40	< 0.001
Natural conception (0–3)	2.30 (0.67)	2.68 (0.55)	− 5.81	< 0.001
PND (0–5)	2.17 (1.32)	3.88 (1.63)	− 8.49	< 0.001
PGT (0–7)	4.97 (1.49)	6.59 (1.13)	− 8.95	< 0.001
Realistic expectations (0–3)	0.75 (0.70)	1.17 (0.92)	− 4.22	< 0.001
Level of deliberation (6–30)	23.36 (4.51)	25.54 (3.61)	− 4.14	< 0.001
Decision self-efficacy (0–100)	76.45 (11.90)	84.50 (12.44)	− 4.80	< 0.001

*N = 48 for T0–T3

### Subgroup analysis

When analyzing the effects of the decision aid for various subgroups on the outcome measures decisional conflict, knowledge, realistic expectations, level of deliberation, and decision self-efficacy, significant effects were found for all outcomes (all *p*s < 0.05). These significant effects were found for males and females, low/middle educated and high educated participants, carriers and partners of carriers, participants with and without a history of cancer, and participants who had had a consultation with a health care provider regarding the reproductive options at baseline or in-between T0 and T3. Participants who had not had a consultation with a healthcare provider at baseline or in-between T0 and T3 showed significant effects reducing decisional conflict and increasing knowledge, realistic expectations, and level of deliberation. No significant effects were found for decision self-efficacy (*p* = 0.478 respectively *p* = 0.190).

A significant effect was found for all outcome measures (all *p*s < 0.05) for participants who plan to have children in the near future (≤ 2 years) whereas no effect was found for realistic expectations (*p* = 0.261), level of deliberation (*p* = 0.095), and decisional self-efficacy (*p* = 0.478) for participants who plan to have children in the more distant future (> 2 years).

For both groups, the confidence in their ability to make a decision was already high at baseline (M = 83.8 and M = 78.1), although their baseline knowledge was relatively low (M = 6.3 and M = 5.2) and increased at T3 (M = 9.0 and M = 9.2). The confidence in their ability to make a reproductive decision among participants with a more distant wish to have children slightly lowered (M = 81.06) and did not significantly increase for participants who had not had a consultation with a healthcare provider at baseline or in-between T0 and T3 (M = 82.5).

## Discussion

Individuals who have a genetic predisposition to cancer and their partners may experience difficulties during reproductive decision-making (Derks-Smeets et al. 2014; Gietel-Habets et al. 2018; Dekeuwer and Bateman 2013; Ormondroyd et al. 2012; Dommering et al. 2010; Donnelly et al. 2013; Van Asperen et al. 2002). Incorporating an online decision aid in reproductive counseling can be helpful for this target group (Derks-Smeets et al. 2014; (de Die-Smulders et al. 2013). In this study, the effects of an online decision aid were assessed 3 months after its use.

Although we lack a concurrent control group, overall results of this study suggest that the decision aid contributes to higher levels of informed decision-making in the oncogenetic counseling setting. T0–T3 comparisons indicated a large and significant overall effect for the outcome measures informed decision-making, decisional conflict, knowledge, realistic expectations, level of deliberation, and decision self-efficacy. These results are in line with previous research indicating reduced decisional conflict, increased knowledge regarding potential options, and facilitation of informed decision-making as possible positive effects of decision aids (O’Connor and Jacobsen 2003; Juraskova et al. 2014; Stacey et al. 2017).

Subgroup analyses indicated no significant effect on decision self-efficacy for participants who wish to have children in the more distant future (> 2 years) and for participants who had not had a consultation with a healthcare provider at baseline or in-between T0 and T3. The increase in knowledge may have contributed to the realization of the complexity of reproductive decision-making. Another possible explanation might be related to the fact that the decision aid is not capable of providing psychological counseling or addressing emotional issues as thoroughly as in an individualized counseling session. Considering the complexity of the decision, for some couples psychological counseling and addressing emotional issues might be a prerequisite to further increase their confidence in making a reproductive decision. For those couples a consultation regarding the reproductive options with a counselor before or after reviewing the decision aid might be needed to further increase their self-efficacy.

Furthermore, although the decision aid resulted in a higher likelihood of informed decision-making, still 42.0% of all participants and 65.5% of the couples did not meet all three conditions for informed decision-making at T3. It is notable that especially participants who wish to have children in the more distant future (> 2 years) did not meet the conditions of sufficient knowledge at T3; about half of these participants still had insufficient knowledge, while the majority of the participants who wish to have children in the near future (≤ 2 years) reported sufficient knowledge. This finding was not reflected in an overall lower engagement among participants with a wish to have children in the more distant future (Reumkens et al. 2018). We therefore tentatively assume that this group is not as engaged yet in reproductive decision-making and therefore may be less committed to making an actual decision compared with those for whom reproductive decision-making is a more pressing issue.

Further, the number of participants meeting all three conditions for informed decision-making at T3 is relatively low compared with other studies (Van den Berg et al. 2006; Lewis et al. 2017; Lo et al. 2017). A possible explanation may lie in the fact that in almost all studies participants received counseling whereas this is not the case for a considerable number of participants in our study. The focus of this paper was also on informed decision-making at the couple level. By definition a couple is less likely to make an informed decision for both partners compared with individuals making personal health decisions. As the decision regarding reproductive options is by nature a dyadic process, pertaining to the interaction between two persons, couples may benefit from the inclusion of methods in the decision aid that further stimulate joint decision-making among partners and facilitate the intra-couple communication during reproductive decision-making. This may contribute to higher levels of joint informed decision-making among our target group. The inclusion of measures of dyadic or joint decision-making would have provided more in-depth information on the interaction and communication processes during reproductive decision-making and the effect of the decision aid on these processes. To our knowledge, currently no validated measurement tools exist to assess such joint decision-making processes. We therefore recommend future research to focus on exploring joint informed decision-making and factors that influence related processes.

An important limitation of this study relates to the use of a pretest-posttest design restricting the internal validity of this study. Maturation and history effects could not be controlled for, and possible interference of other factors, such as use of other information sources, cannot be excluded. However, the effects are probably mainly due to the use of the decision aid as the effects were measured immediately after use of the DA. This minimizes the risk of possible bias of other factors such as counseling or retrieval of additional information sources. Despite of the fact that sample sizes are overall relatively small in reproductive genetics, future studies should strive to use an experimental design such as a randomized control trial (RCT) which is capable of providing the strongest level of evidence. Furthermore, this study included mainly participants who were well-educated. Although this is in line with general characteristics of oncogenetic counselees (van der Giessen et al. 2017), future research should strive to recruit patients with lower educational levels and lower levels of health literacy and determine effects of decisional support among these subgroups. It was also not feasible to collect information about non-participants. Therefore, we were unable to calculate a response rate, nor report on their reasons for non-participation. Additionally, the dropout rate was 29% and the reasons for dropout are unknown. Ideally, one should have information into the reasons for non-responders and dropout as this might give indications for improvement of the decision aid and the willingness of the target population to use the decision aid. However, in practice it is difficult to get this information.

In conclusion, our findings suggest that the online decision aid is an appropriate tool to effectively supplement standard reproductive counseling to support persons having a genetic predisposition to cancer and their partners in making an informed reproductive decision. In order to make wider use of the tool possible, the decision aid can be adapted to fit the needs of couples with other hereditary conditions.

Several positive outcomes indicative of informed decision-making were found 3 months after use of the decision aid. Therewith use of the decision aid may lessen the negative psychological impact of decision-making on couples’ daily life and wellbeing. The decision aid is an appropriate tool to be used in addition to reproductive counseling to support couples who are in need of reproductive decision support. Currently we are conducting an explorative implementation study to clarify optimal timing of providing the decision aid and how to best incorporate the decision aid in daily practice.

## Data Availability

The datasets used and/or analyzed during the current study are available from the corresponding author on reasonable request.
